# Combined ^18^F-FET PET and diffusion kurtosis MRI in posttreatment glioblastoma: differentiation of true progression from treatment-related changes

**DOI:** 10.1093/noajnl/vdab044

**Published:** 2021-03-10

**Authors:** Francesco D’Amore, Farida Grinberg, Jörg Mauler, Norbert Galldiks, Ganna Blazhenets, Ezequiel Farrher, Christian Filss, Gabriele Stoffels, Felix M Mottaghy, Philipp Lohmann, Nadim Jon Shah, Karl-Josef Langen

**Affiliations:** 1 Institute of Neuroscience and Medicine, Research Centre Juelich, Juelich, Germany; 2 Department of Neuroradiology, Circolo Hospital and Macchi Foundation, Varese, Italy; 3 Department of Neurology, Faculty of Medicine and University Hospital Cologne, University of Cologne, Cologne, Germany; 4 Center for Integrated Oncology (CIO), Universities of Aachen, Bonn, Cologne and Duesseldorf, Germany; 5 Department of Nuclear Medicine, Medical Center—University of Freiburg, Faculty of Medicine, University of Freiburg, Freiburg, Germany; 6 Department of Nuclear Medicine, RWTH Aachen University, Aachen, Germany; 7 Department of Radiology and Nuclear Medicine, Maastricht University Medical Center (MUMC+), Maastricht, The Netherlands; 8 Department of Stereotaxy and Functional Neurosurgery, Faculty of Medicine and University Hospital Cologne, University of Cologne, Cologne, Germany; 9 Department of Neurology, RWTH Aachen University, Aachen, Germany; 10 JARA—BRAIN—Translational Medicine, Aachen, Germany

**Keywords:** amino acid PET, brain tumor, diffusion kurtosis MRI, pseudoprogression, tumor progression

## Abstract

**Background:**

Radiological differentiation of tumor progression (TPR) from treatment-related changes (TRC) in pretreated glioblastoma is crucial. This study aimed to explore the diagnostic value of diffusion kurtosis MRI combined with information derived from *O*-(2-[^18^F]-fluoroethyl)-l-tyrosine (^18^F-FET) PET for the differentiation of TPR from TRC in patients with pretreated glioblastoma.

**Methods:**

Thirty-two patients with histomolecularly defined and pretreated glioblastoma suspected of having TPR were included in this retrospective study. Twenty-one patients were included in the TPR group, and 11 patients in the TRC group, as assessed by neuropathology or clinicoradiological follow-up. Three-dimensional (3D) regions of interest were generated based on increased ^18^F-FET uptake using a tumor-to-brain ratio of 1.6. Furthermore, diffusion MRI kurtosis maps were obtained from the same regions of interest using co-registered ^18^F-FET PET images, and advanced histogram analysis of diffusion kurtosis map parameters was applied to generated 3D regions of interest. Diagnostic accuracy was analyzed by receiver operating characteristic curve analysis and combinations of PET and MRI parameters using multivariate logistic regression.

**Results:**

Parameters derived from diffusion MRI kurtosis maps show high diagnostic accuracy, up to 88%, for differentiating between TPR and TRC. Logistic regression revealed that the highest diagnostic accuracy of 94% (area under the curve, 0.97; sensitivity, 94%; specificity, 91%) was achieved by combining the maximum tumor-to-brain ratio of ^18^F-FET uptake and diffusion MRI kurtosis metrics.

**Conclusions:**

The combined use of ^18^F-FET PET and MRI diffusion kurtosis maps appears to be a promising approach to improve the differentiation of TPR from TRC in pretreated glioblastoma and warrants further investigation.

**Key Points**

- Diffusion kurtosis MRI in FET PET-positive areas provides 88% diagnostic accuracy in recurrent gliomas.
- Combination of diffusion kurtosis MRI and FET PET increases diagnostic accuracy to 94%.

**Importance of the Study**

The differentiation of tumor progression from treatment-related changes in patients with pretreated glioblastoma is difficult based on structural MRI alone. This study explores the combination of PET using the amino acid tracer *O*-(2-[^18^F]-fluoroethyl)-l-tyrosine (FET) and microstructural information obtained from diffusion kurtosis imaging (DKI). Both these methods may provide additional information for this clinically important question. The combination of FET PET and DKI resulted in high diagnostic accuracy, warranting further investigations. The developed FET/DKI index offers a simple way to apply this approach in clinical practice.

Due to its aggressive infiltrative growth and the high relapse rate, glioblastoma is the most lethal brain tumor in adults and despite maximal treatment, only 5% of patients with glioblastoma survive for 5 years or longer following diagnosis.^1^ The diagnosis of tumor progression (TPR) based on standard MRI alone is challenged by the necessity to differentiate TPR from non-neoplastic, treatment-related changes (TRC), such as pseudoprogression or radiation necrosis.^2^ In particular, pseudoprogression manifests itself as progressive or newly contrast-enhancing lesions, usually within 3 months after completion of radiotherapy in 15–30% of patients with malignant glioma.^3^ TRC often mimic TPR and consequently tend to interfere with day-to-day patient care, representing a critical clinical dilemma.^4^ Despite the considerable efforts already made in improving response assessment and supporting clinical decision-making,^5^ the further development of reliable imaging biomarkers for more accurate diagnostics of TRC remains urgently needed.

Contrast-enhanced MRI is the method of choice in neuro-oncology, playing a key role in diagnostics and the assessment of treatment response.^6^ However, conventional MRI has a limited specificity in differentiating TPR from TRC,^4^ and a major disadvantage of anatomic MRI is the lack of metabolic information. In contrast, PET using radiolabelled amino acids, such as *O*-(2-[^18^F]-fluoroethyl)-l-tyrosine (^18^F-FET), is a sensitive tool for imaging metabolic tumor activity and can provide additional information for improved diagnostics.^7^ The Response Assessment in Neuro-Oncology (RANO) working group and the European Association of Neuro-Oncology advocate the use of amino acid PET as a complementary tool to MRI in the management of patients with brain tumors.^8^ In particular, ^18^F-FET PET has been recently shown to facilitate improved differentiation of TRC from TPR with high diagnostic accuracies, in the range of 80–90%.^9,10^

The establishment of advanced MRI techniques represents another rapidly developing field relevant for neuro-oncology,^7^ with diffusion MRI, which is sensitive to microstructural cellular tissue architecture, being increasingly used in the assessment of brain tumors.^11^ The apparent diffusion coefficient derived from diffusion-weighted or diffusion tensor imaging (DTI)^12^ tends to inversely correlate with tumor cellularity^13^ and was tested in treatment response assessment^14^ alongside the discrimination of TRC from TPR.^15^ However, the reliable use of the mean DTI metrics in neuro-oncology is often limited due to intratumor microenvironmental heterogeneity,^16^ the hallmark of most brain malignancies. On the other hand, histogram analysis of DTI metrics enables the consideration of more detailed information and has been shown to provide added value in grading gliomas^17^ and in treatment monitoring.^18–20^

Further perspectives concerning diffusion MRI in brain tumors are associated with the current development of novel multi-shell diffusion techniques,^11,21^ employing high *b* values beyond the typical DTI range (≤1 μm^2^/ms). These techniques address the non-Gaussian diffusion properties of water in tissue and encode additional microstructural features. In particular, diffusion kurtosis imaging (DKI)^22^ enables the estimation of diffusion tensor (DT) and specific kurtosis tensor (KT) metrics from the same measurement, at clinically practicable acquisition times. Consequently, it is attracting growing interest in neuroradiology, with applications reported for stroke, neurodegenerative diseases, and tumors,^11,21,23^ and promising biomarkers have been found for assessing glioma grades and cellular proliferation.^24,25^

A small number of studies have shown the potential of combining ^18^F-FET PET with diffusion and/or other MRI methods to examine brain tumor patients^26,27^ with the help of modern hybrid PET/MRI technologies.^28^ There is also growing evidence that the use of a combination of structural and advanced MRI modalities alongside ^18^F-FET PET can considerably enhance the diagnostic accuracy for the detection of TPR and predicting response to treatment.^3,27,29,30^

We hypothesize that lesions showing slightly abnormal or increased ^18^F-FET uptake in the area of suspected TPR might be helpful for identifying areas with abnormal microstructural properties, which could then be further differentiated using advanced diffusion MRI modalities. The purpose of this exploratory hybrid PET/MRI study was to evaluate DT and KT parameters in lesions with increased ^18^F-FET uptake and to assess their accuracy in differentiating TPR and TRC using histogram analysis.

## Materials and Methods

### Patients

From February 2013 to March 2016, 32 patients with histopathologically proven glioblastoma and clinical signs or MRI findings suggestive of TPR based on the RANO^8^ criteria were retrospectively included in the study. All patients were examined using a hybrid PET/MRI scanner and fulfilled the following inclusion criteria: (1) hybrid ^18^F-FET PET/MRI scan with DKI and structural MRI brain tumor imaging protocol, (2) prior tumor resection or biopsy followed by temozolomide (TMZ) chemoradiation, and (3) repeat biopsy or clinical follow-up data and imaging follow-up data available from at least 6 months after the hybrid PET/MRI investigations. The local ethics committee approved the retrospective analysis of the data. There was no conflict with the Declaration of Helsinki. Before imaging, all patients had given written informed consent for the PET and MRI investigation and the use of the acquired data for scientific purposes. Further details on the patient cohort are presented in Supplementary Table S1.

The diagnosis of TRC or TPR was based on the criteria defined by Young et al.^31^ If no-repeat histopathology was available (in 20 patients), the clinical diagnosis of TRC or TPR was reached via consensus of 2 experienced neurooncologists based upon a complete chart review and review of follow-up MRI. The diagnosis of TRC was assumed if no change in treatment was required for at least 6 months after PET/MRI.

### ^18^F-FET PET Imaging and Data Analysis

All patients were scanned using a high-resolution 3T hybrid PET/MRI scanner (BrainPET, Siemens Healthcare, axial field of view, 19.2 cm). Image data were corrected for random and scatter coincidences, as well as dead time, prior to OP-OSEM reconstruction provided by the manufacturer (2 subsets, 32 iterations). The reconstructed dynamic data set consisted of 16 time frames (5 × 1 min; 5 × 3 min; 6 × 5 min). Since the hybrid PET/MRI scanner does not provide a transmission source, attenuation correction was performed with a template-based approach using MRI.^30^

^18^F-FET PET data were applied and analyzed as described previously.^32^ In brief, dynamic PET studies were acquired for 50 min after intravenous injection of approximately 3 MBq ^18^F-FET/kg of body weight. Mean ^18^F-FET uptake in the tumor was determined by a two-dimensional (2D) auto-contouring process using a tumor-to-brain ratio (TBR) of at least 1.6 in the summed ^18^F-FET PET images from 20 to 40 min post-injection. For calculating the maximal amino acid uptake, a circular region of interest (ROI) with a diameter of 1.6 cm was centered on the maximal tumor uptake. Mean and maximum TBRs (TBR_mean_ and TBR_max_) were calculated by dividing the mean and maximum standardized uptake value (SUV) of the tumor ROIs by the mean SUV of healthy brain tissue. Time-activity curves (TACs) of ^18^F-FET uptake in the tumor were obtained by applying a spherical volume of interest with a volume of 2 ml (diameter 1.6 cm) to the entire dynamic dataset. Time-to-peak values, derived from the TACs (TTP; minimum from the beginning of the dynamic acquisition up to the maximum SUV of the lesion) and the slope of the TAC of ^18^F-FET uptake, were calculated by fitting a linear regression line to the late phase of the curve (20–50 min post-injection). The slope was expressed in the change of SUV per hour.

### MRI Acquisition Protocol

The MRI protocol used with the hybrid PET/MRI scanner included a T_1_-weighted magnetization-prepared rapid gradient echo (MP-RAGE) sequence, a T_2_-weighted fluid-attenuated inversion recovery sequence, and a contrast-enhanced T_1_-weighted MP-RAGE sequence (CE-T_1_) conducted following the injection of the contrast agent, gadoteric acid (Dotarem; Guerbet), with a dose of 0.1–0.2 mmol/kg of body weight.^33^ The diffusion MRI sequence had the following acquisition parameters: TR/TE = 9700/105 ms; bandwidth = 1594 Hz/px; *b* values = 0, 1000, 2500 s/mm^2^; number of averages = 2; number of gradient directions = 30; voxel size = 2 × 2 × 2 mm^3^; matrix size = 112 × 112 × 68.

### DKI Image Processing

Diffusion-weighted MRI images were corrected for eddy current distortions and head motion using the tool “eddy-correct” available in FSL; gradient field directions were reoriented^34^ using in-house Matlab scripts (R2014a, 2014, The Mathworks Inc.). Positive bias in the signal was corrected using the power images method,^35,36^ with the standard deviation of the background noise estimated using the approach by Aja-Fernández et al.^37^ and further regularized using ExploreDTI.^38^ DKI analysis was performed using the nonlinear least-squares estimator available in ExploreDTI. Finally, both DT (mean/radial/axial diffusivities, MD/RD/AD) and specific KT (mean/radial/axial kurtoses, MK/RK/AK) metrics were evaluated using ExploreDTI.

Image co-registration and tumor segmentation were carried out with the Brain PET/MRI analysis tool of the PMOD software (version 3.707, 2015, PMOD Technologies Ltd) by board-certified radiologists (F.M. and C.F.). PET images were co-registered via rigid matching with normalized mutual information using a Gaussian smoothing algorithm applied to the contrast-enhanced T_1_-weighted 3-dimensional (3D) MRI. DT/KT parameter maps were subsequently registered to CE-T_1_ via affine rigid body registration with normalized mutual information as a cost function. 3D ROIs were semi-automatically segmented on the PET images using a TBR threshold of 1.6.^39^ The corresponding voxel-by-voxel-based 3D ROIs were thereafter extracted for all DT/KT metrics from parameters maps and used for histogram generation. 2D ROIs were drawn on DT/KT parameter maps in the contralateral normal-appearing white matter, at the level of the centrum semiovale, avoiding CSF spaces and blood byproducts when present. Two readers (F.D. and G.B.) evaluated the accuracy of co-registrations and the absence of visible distortions.

### Histogram Analysis of DT and KT Metrics

The histogram analysis was performed for DT/KT 3D ROI datasets using Matlab and in-house scripts. Voxel intensity values outside the 0.5–3.5 μm^2^/ms range for DT maps and outside the 0.4–1.2 range for KT maps were discarded in order to reduce the influence of noisy points. Relative frequency histograms were generated for each subject after normalization of the individual voxel-per-bin histograms by the volume of the corresponding 3D ROI. Histogram smoothing was performed using a moving average window of 0.14 μm^2^/ms for DT and 0.04 for KT histograms. Histogram means and centiles (C5, C10 for DT and C90, C95 for KT maps) were extracted from each individual subject’s histogram, thus providing 18 DT/KT variables in total. The normalization of the histogram parameters to the values in contralateral normal-appearing white matter ROIs was omitted since intergroup differences in these regions were not significant (Supplementary Table S2).

### Statistical Analysis

Statistical analysis was performed using the software MedCalc (c17.2, MedCalc Software Bvba, 2017). The normal distribution of variables was assessed using the Kolmogorov–Smirnov test. Intergroup differences in the mean values of DT/KT parameters relating to 2D ROIs in normal-appearing white matter, 3D ROI histogram parameters of DT/KT metrics, and ^18^F-FET PET parameters (TBR_max_, TBR_mean_, TTP, slope) were compared using the Mann–Whitney U test.

3D ROI histogram parameters of DT/KT metrics were considered significant for *P* ≤ .0028 = .05/18, where the value of *α* was set using Bonferroni correction for multiple comparisons (*n* = 18). Comparisons of 4 ^18^F-FET PET parameters were considered significant for *P* ≤ .0125 = .05/4. Given the exploratory nature of this work and the restrictive nature of the Bonferroni correction, we additionally regarded non-corrected *α* = 0.05 and refer to the findings with 0.003 < *P* ≤ .05 for DT/KT metrics and with 0.0125 < *P* ≤ .05 as “significant prior to correction.” ^40^ Possible associations between the variables were ignored as the primary goal of this study was to identify the most promising biomarkers.

Receiver operating characteristic (ROC) curves were employed to assess the areas under the ROC curve (AUC) and to find optimal cutoff values of the histogram parameters. Only parameters that were statistically significant in intergroup comparisons were considered. Statistically significant differences among AUCs were explored using the DeLong methodology with an exact binomial 95% confidence interval.

Univariate logistic regressions with “diagnosis” as the dependent binary variable were used to test the predictive ability of the DT/KT histogram parameters that were significant according to the Mann–Whitney U test results. Finally, in order to identify parameter combinations that can more accurately predict TPR compared to individual parameters, a multivariate logistic regression model was constructed with diagnosis as the dependent variable. The regression coefficients for MK C90 (4.7) and TBR_max_ (39.2) were further used as weighting factors in order to produce a single, clinically applicable FET–DKI index for the differentiation between TPR and TRC in the following way:

$$\begin{array}{l} FET-DKI\;index\;=\;4.7\;\times \;TBR_{\max}+\;39.2\;\times \;MKC90. \end{array}$$ (1)

## Results

Eleven patients were allocated to the TRC group and 21 patients to the TPR group. The diagnosis was based on histopathology in 12 cases and on clinical follow-up in 20 cases. One patient from the TRC group had a history of recurrence prior to PET/MRI, while 11 TPR patients experienced one or more recurrences. The whole cohort underwent surgery and concurrent TMZ chemoradiation therapy plus adjuvant TMZ as first-line therapy. Second-line bevacizumab (BEV) was employed alone or in combination with other agents in 3 patients. At the time of PET/MRI, 17 patients were being treated with TMZ, 1 patient from the TPR group was treated with BEV, and 14 patients were off therapy (Supplementary Table S1).

Statistically significant (*P* < .0028) increases were found for all histogram parameters of the KT metrics in the TPR compared to the TRC group, except for AK C90 and AK C95 (which were significant prior to correction, *P* ≤ .05). The histogram parameters of the DT metrics did not reach statistical significance (*P* > .008) after Bonferroni correction. However, it is worth pointing out that DT metrics showed a clear tendency to be lower in the TPR group and 4 of them (mean RD, RD C5, RD C10, and MD C10) were significant prior to correction (*P* ≤ .05). There was no significant difference in ^18^F-FET PET parameters TBR_mean_, TTP, and slope between the groups, whereas TBR_max_ was significantly lower in TRC. Table 1 provides a summary of the medians and interquartile range evaluated for the 3D ROI histogram parameters of the DT/KT and for the ^18^F-FET PET parameters for both groups, along with *P* values of the intergroup comparisons.

**Table 1.** Comparison of DT/KT Metrics (Upper Panel) and ^18^F-FET PET Parameters (Bottom Panel) in the TRC and TPR Groups

Parameter		TRC (*n* = 11)	TPR (*n* = 21)	*P*
MD	Mean	1.54 (1.42/1.7)	1.32 (1.24/1.55)	.060
	C5	1.00 (0.94/1.10)	0.93 (0.80/1.02)	.065
	C10	1.13 (1.05/1.18)	1.02 (0.87/1.1)	.023*
RD	Mean	1.54 (1.31/1.63)	1.21 (1.13–1.43)	.034*
	C5	0.92 (0.85/1.00)	0.80 (0.66/0.86)	.020*
	C10	0.99 (0.96/1.10)	0.87 (0.73/0.94)	.008*
AD	Mean	1.84 (1.69/1.91)	1.57 (1.46/1.83)	.100
	C5	1.24 (1.13/1.30)	1.14 (0.96/1.30)	.197
	C10	1.32 (1.20/1.37)	1.20 (1.03/1.35)	.171
MK	Mean	0.50 (0.48/0.58)	0.61 (0.57/0.69)	.002**
	C90	0.61 (0.55/0.73)	0.78 (0.70/0.83)	.001**
	C95	0.68 (0.57/0.77)	0.82 (0.76/0.90)	.001**
RK	Mean	0.52 (0.50/0.60)	0.63 (0.58/0.68)	.002**
	C90	0.65 (0.57/0.76)	0.81 (0.73/0.88)	.002**
	C95	0.74 (0.59/0.80)	0.88 (0.78/0.98)	.002**
AK	Mean	0.51 (0.48/0.55)	0.59 (0.55/0.65)	.002**
	C90	0.62 (0.56/0.68)	0.73 (0.67/0.79)	.003*
	C95	0.68 (0.61/0.76)	0.78 (0.72/0.86)	.015*
[^18^F]-FET PET	TBR_mean_	2.00 (1.83/2.08)	2.00 (1.90/2.23)	.200
	TBR_max_	2.50 (2.13/3.20)	3.30 (2.75/3.93)	.012**
	TTP	37.50 (37.5/27.5)	27.50 (32.5/22.5)	.067
	Slope	0.42 (0.65/0.32)	0.25 (0.61/0.03)	.210

3D ROI histogram parameters (the means and the centiles) of the DT/KT metrics and FET PET parameters are presented by their medians and quartiles (Q1/Q3 in parentheses) and by *P* values of the intergroup comparisons. Diffusivities are given in units of μm^2^/ms. Significant (Bonferroni corrected) comparisons are indicated by **, *suggestively* significant (non-corrected) by *.

**Figure 1.**
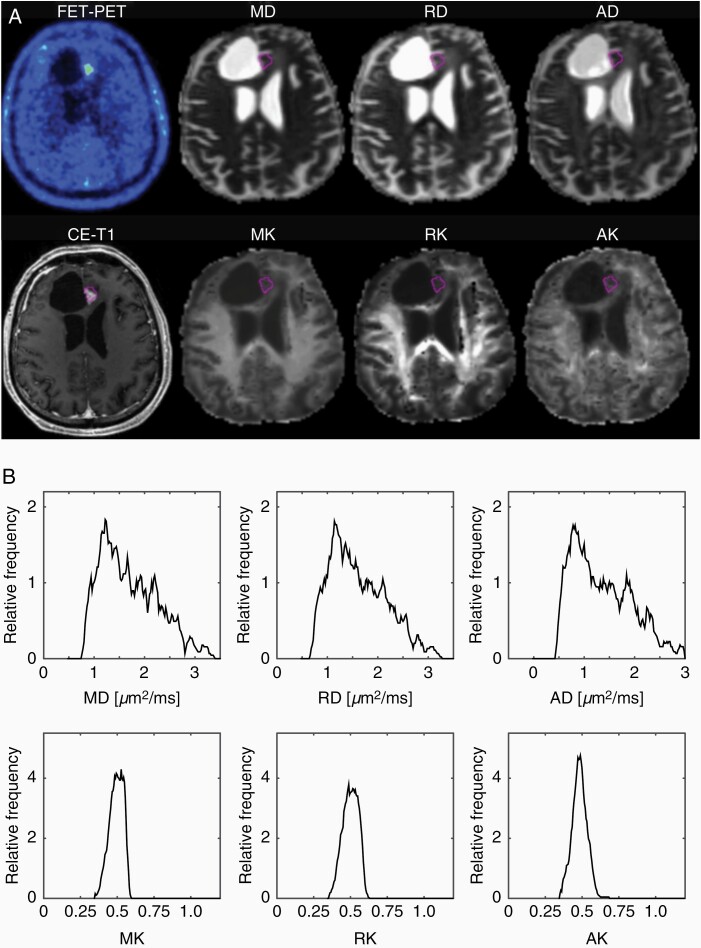
Examples of ^18^F-FET uptake, CE-T_1_ MRI, and DT/KT parameter maps (A), and the histograms corresponding to the ROIs highlighted in the maps for a patient with TRC (B).

**Figure 2.**
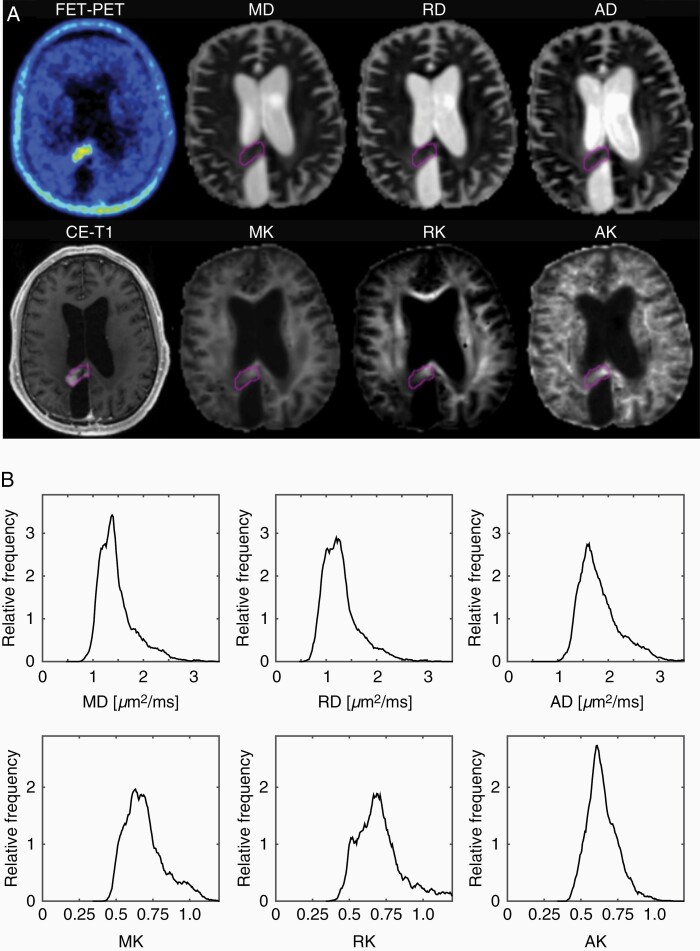
Examples of ^18^F-FET uptake, CE-T_1_ MRI, and DT/KT parameter maps (A), and the histograms corresponding to the ROIs highlighted in the maps for a patient with TPR (B).

**Figure 3.**
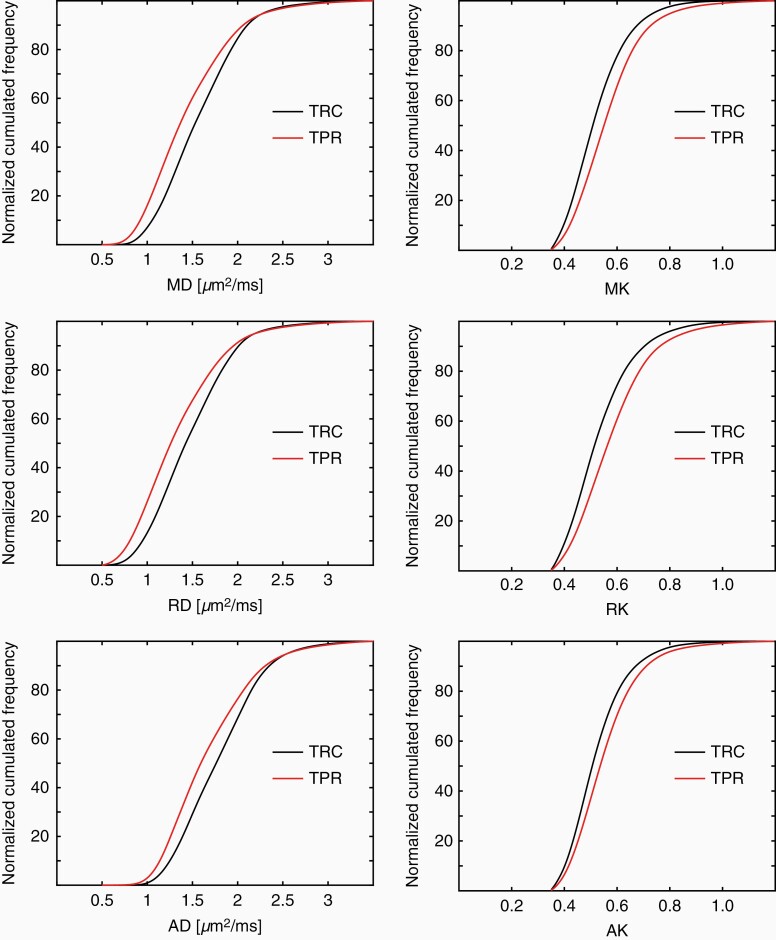
Examples of cumulative relative frequency histograms of DT (MD/RD/AD) and KT (MK/RK/AK) parameters showing shifts toward *lower* diffusivity values and *higher* diffusion kurtosis values in the TPR compared to the TRC group.

Examples of ^18^F-FET uptake, CE-T_1_, and DT/KT parameter maps and the histograms corresponding to the ROIs highlighted in the maps for one representative patient from each of the TRC and TPR groups are shown in Figures 1 and 2, respectively. In the TRC patient (Figure 1), diffusivity histograms constitute larger areas with higher values, whereas KT histograms are clearly shifted toward lower values in comparison to the patient from the TPR group (Figure 2). Accordingly, at the group level, the cumulative relative frequency histograms of the DT/KT parameters in the TPR group appear shifted toward *lower* diffusivities and *higher* diffusion kurtoses compared to the TRC group (Figure 3).

The MK C90 cutoff value of 0.62 yielded the largest AUC = 0.87 (*P* < .0001), with a sensitivity of 100%, a specificity of 64%, and an accuracy of 88%. A sensitivity of 100% was also reached by MK mean and RK C90 showing AUCs of 0.85 (*P* < .0001) and 0.83 (*P* = .0001), respectively. The highest specificity (91%) was observed in MK C95 with an AUC of 0.86 (*P* < .0001). AUCs were not significantly different between KT parameters (*P* > .05). ROC analysis for TBR_max_, the only significant parameter among the ^18^F-FET PET metrics, yielded a cutoff of 2.95 and an AUC of 0.77 (*P* = .003) with a sensitivity of 71%, a specificity of 73%, and an accuracy of 72%.

**Figure 4.**
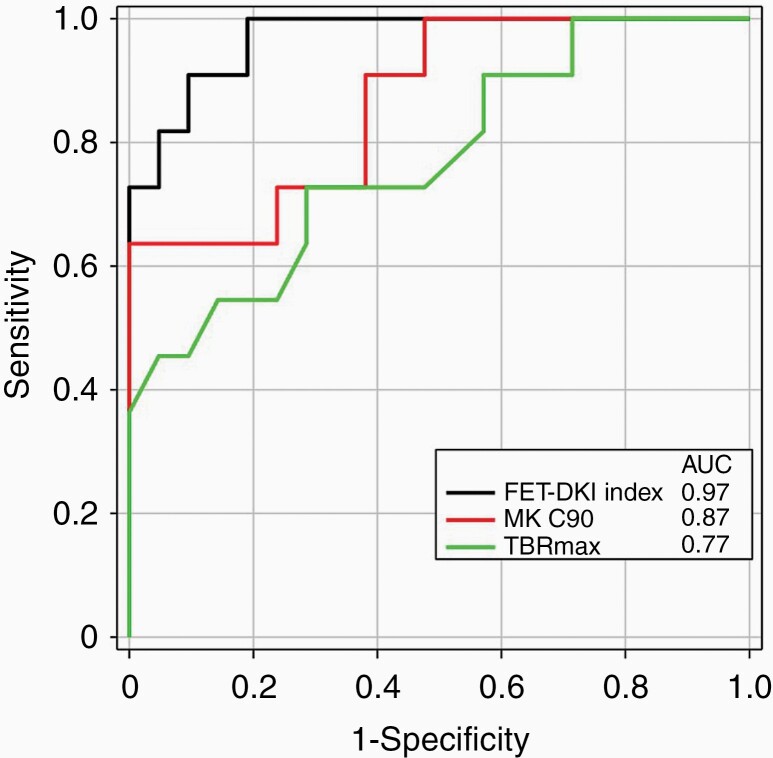
ROC analysis for differentiation between the TPR and TRC groups based on MK C90 (red), TBR_max_ (green), and FET–DKI index (black). AUCs are indicated in the figure legend.

The multivariate logistic regression model was tested for a combination of metrics, with the highest correct classification rates in each of the DKI and ^18^F-FET PET parameter sets (ie, MK mean, MK C90, and TBR_max_), and for various combinations of DKI and ^18^F-FET PET parameters that were significant in intergroup comparisons. However, as none of the latter combinations yielded better diagnostic accuracy than the combination of MK C90 and TBR_max_, these tests were not pursued in further analysis. The combination of MK C90 and TBR_max_ resulted in the highest diagnostic accuracy (94%) with an AUC of 0.97 (Table 2, bottom panel). The ROC analysis of the single FET–DKI index (Eq. 1) yielded a cutoff of more than 41 to identify TPR resulting in an AUC of 0.97, a sensitivity of 95%, a specificity of 91%, and an accuracy of 94% for differentiation between TPR and TRC. Table 2 summarizes the results of the ROC curve and logistic regression analysis for the histogram parameters of the KT metrics and TBR_max_ of ^18^F-FET uptake. The results of the ROC analysis for differentiation between the TPR and TRC groups are visualized in Figure 4 for MK C90 (red), TBR_max_ (green), and FET–DKI index (black) for comparison.

**Table 2.** Diagnostic Accuracy of the KT Histogram Parameters and TBR_max_ of FET PET for Differentiating Between the TRC and TPR as Assessed by the ROC and Univariate Logistic Regression Analysis (Upper Panel) and the Multivariate Logistic Regression (Bottom Panel): AUC, cutoff values, 95% CI values in parentheses, sensitivity, specificity, and *P* values

		AUC	95% CI	Cutoff (>)	Sensitivity	Specificity	Accuracy	*P*
MK	Mean	0.85	0.68–0.95	0.51	100	64	88	<.0001
	C90	0.87	0.70–0.96	0.62	100	64	88	<.0001
	C95	0.86	0.69–0.95	0.79	67	91	75	<.0001
RK	Mean	0.83	0.66–0.94	0.56	90	70	84	.0002
	C90	0.83	0.66–0.94	0.67	100	64	88	.0001
	C95	0.77	0.66–0.94	0.77	81	73	78	<.0001
AK	Mean	0.83	0.67–0.95	0.56	90	64	81	<.0001
^18^F-FET PET	TBR_max_	0.77	0.59–0.90	2.95	71	73	72	.003
FET–DKI index	TBR_max_ + MK C90 (weighted)	0.97	0.89–1.02	41	95	91	94	<.0001
FET–DKI	TBR_max_ and MK C90	0.97	0.83–0.99	—	—	—	91	<.0001

## Discussion

The main finding of our exploratory study shows that exploiting combined information relating to amino acid transport by ^18^F-FET PET and microstructural information from diffusion MRI may offer synergistic benefits for clinical practice when addressing the issue of discrimination between TPR and TRC. That is to say, for patients with suspected recurrent glioblastoma, areas of the brain with increased ^18^F-FET uptake exhibit microstructural differences, which can be captured by KT histogram parameters, enabling the differentiation of TPR and TRC with high diagnostic accuracy. Another important finding is that the KT metrics in the areas with high ^18^F-FET uptake essentially outperformed DT metrics in differentiating TPR and TRC. This finding is in line with the previously reported higher sensitivity of KT metrics in the characterization of various pathological brain states.^41,42^ Furthermore, multivariate logistic regression analysis indicated that the differentiation between TPR and TRC could be improved by a combination of KT histogram parameters and TBR_max_. Based on this result, we generated a single robust FET–DKI index by summing up weighted values of TBR_max_ and MK C90 (Eq. 1). The FET–DKI index allowed for the differentiation of TPR and TRC with an AUC of 0.97 at a cutoff of 41 and, thus, might be useful in clinical decision making. We would like to emphasize that the high performance of the KT metrics and the combined FET–DKI approach observed in this work can be attributed, on the one hand, to the segmentation of the lesions by areas with increased ^18^F-FET uptake rather than on contrast-enhanced MRI and, on the other hand, to the application of the histogram analysis, reducing averaging effects of either TPR or treatment-modulated lesion heterogeneity.

Our results concerning DT/KT parameters require discussion in the context of the current literature. Regarding the normal-appearing white matter, the values of diffusivities and diffusion kurtoses observed in this work were in the range of those seen in previous investigations.^24,25^ To date, only a small number of studies have applied the DKI histogram analysis to patients with brain tumors.^43,44^ These studies, however, have focused primarily on glioma grading, so that a direct comparison with our results is not possible. Nevertheless, several studies have explored the role of conventional DTI in differentiating TPR from TRC^30,45,46^ with the majority of data indicating insufficient diagnostic accuracy. In our study, a clear tendency for lower diffusivity values in the TPR group was observed; however, none of the DT metrics reached statistical significance in the intergroup differentiation.

A positive correlation between kurtosis metrics and Ki-67, a marker of cellular proliferation, was found by Jiang et al.,^25^ suggesting that higher cellularity in conjunction with other cellular, subcellular, and extracellular changes contributes to the increasing microstructural complexity of tumorous tissue. Similarly, Hempel et al.^41^ recently found significant variations in MK according to molecular subtypes of gliomas with well-known differences in histological features. More generally, it is suggested that with the exception of the most straightforward quantitative parameters, such as cell density, various additional features (cell size, shape, nucleoplasm to cytoplasm ratio, etc.) of neoplastic cells^47^ and extracellular matrix composition^48^ may also influence water diffusion.

The results indicate that posttreatment multicompartmental disruption of microenvironment^49^ results in globally lower diffusion kurtosis in TRC compared to the tightly organized axon bundles of normal-appearing white matter. In contrast, in TPR, proliferating and invading/infiltrating cells and extracellular space tortuosity result in higher kurtosis than in TRC, yet not as high as in normal-appearing white matter. A variable mixture of features suggesting TPR and TRC are routinely reported at histology.^49^ In this regard, our novel ^18^F-FET PET guided ROI definition approach allowed us to focus on metabolically active tissue, thereby discarding “pure” vasogenic perilesional edema and nonspecific blood–brain barrier disruption while including the surrounding normal-appearing parenchyma on MRI which, however, showed above threshold ^18^F-FET uptake on PET imaging. Furthermore, since the diffusion signal may fluctuate during and after therapy, leading to confusing interpretations when measured at a single time point,^18,48^ the metabolic information provided by ^18^F-FET PET may also help in reducing confounding factors.

In the present study, an ^18^F-FET uptake threshold of 1.6 or more above background was used to identify metabolically abnormal tissue. This threshold was determined in untreated gliomas to best differentiate glioma tissue from peritumoral tissue.^8^ In the post-therapeutic situation, however, reactive changes in the tissue, such as reactive astrocytosis, lead to a moderate increase of ^18^F-FET uptake so that the 3D ROI used in this study probably includes both TPR and TRC. Nevertheless, ^18^F-FET PET has shown good diagnostic accuracy in discriminating between TRC and TPR.^9,29^ Our results are in agreement with those published,^9,29^ although we did not find significant differences for TBR_mean_, which might be attributed to the relatively small sample size in our study.

The limitations of this work refer to the relatively small number of patients, the absence of a validation set, the monocentric approach, a small number of biopsies in the TRC group, a wide range of timings after various treatments all of which may hinder the generalizability of the results. Furthermore, anti-angiogenic drugs or corticosteroids might have an influence especially on contrast enhancement in MRI, that is, blood–brain barrier permeability. However, the uptake of ^18^F-FET that was used for ROI definition is independent of BBB disruption. Hence, the influence of these drugs on the results is unlikely. Moreover, there is a selection bias, because only patients are referred for ^18^F-FET PET in whom the diagnosis is unclear on the basis of conventional MRI and clinical parameters. Thus, the collective is representative of the problematic cases in clinical practice, which strengthens the validity of the results. Nevertheless, further studies with neuropathological validation of neuroimaging findings in a higher number of patients are warranted to confirm our findings.

In conclusion, our results indicate that the combined use of amino acid PET using the tracer ^18^F-FET and KT histogram analysis may help to pinpoint the differences between TPR and TRC. KT histogram analysis has been demonstrated to have a potential value in defining the subtle imaging differences between progressive glioblastoma and TRC and performs better than DT metrics. Higher diagnostic accuracy was obtained by combining ^18^F-FET PET (TBR_max_) and the higher-end KT histogram centiles (MK C90) in comparison to stand-alone ^18^F-FET PET and DT/KT metrics. Thus, the combined analysis of amino acid PET and advanced MRI provides more diagnostic information than either modality alone. Future studies should look for diffusion abnormalities within tumor subregions of highest ^18^F-FET uptake and make comparisons with histomolecular findings in order to shed more light on their relationship with the underlying microstructural and morphological features of the most cell-dense tumor habitat.

## Funding

None declared.

## Supplementary Material

vdab044_suppl_Supplementary_Table_S1

vdab044_suppl_Supplementary_Table_S2
