# Supplementary material for: Combined 18F-FET PET and diffusion kurtosis MRI in posttreatment glioblastoma: differentiation of true progression from treatment-related changes
Source: Neurooncol Adv. 2021 Mar 10;3(1):vdab044. doi: 10.1093/noajnl/vdab044 (PMC8117449; doi:10.1093/noajnl/vdab044)
Supplement: vdab044_suppl_Supplementary_Table_S1 [file vdab044_suppl_supplementary_table_s1.docx]

Table 1S

**Patients Characteristics and Clinical Data**

|  | TRC^1^ (n=11) | TPR^2^  (n=21) | Total  (n=32) |
| --- | --- | --- | --- |
| Age: group mean (range) | 58 (32-64) | 50 (26-74) | 52 (26-74) |
| Gender (M ; F) | 5 ; 6 | 14 ; 7 | 19 ; 13 |
| Lesion volume (cm^3^): median (range) | 4 (2/20) | 3 (2/18) | 3.5 (1/55) |
| Diagnosis based on repeat histopathology | 2 | 10 | 12 |
| Diagnosis based on clinical follow-up | 9 | 11 | 20 |
| Initial treatment |  |  |  |
| Resection + CCRT^3^ + adjuvant TMZ^4^ | 11 | 21 | 32 |
|  |  |  |  |
| Number of recurrences |  |  |  |
| None | 9 | 13 | 22 |
| First | 2 | 5 | 7 |
| Second | 0 | 2 | 2 |
| Third | 0 | 1 | 1 |
| Treatment at last recurrence |  |  |  |
| Surgery only | 1 | 1 | 2 |
| BEV^5^ alone or combined with other agents | 0 | 3 | 3 |
| TMZ alone or combined | 0 | 3 | 3 |
| Second line CCRT + adjuvant TMZ | 0 | 1 | 1 |
| Radiation therapy | 1 | 0 | 1 |
| Therapy at time of PET/MRI |  |  |  |
| TMZ | 6 | 11 | 17 |
| BEV alone or combined with Lomustine | 0 | 1 | 1 |
| no therapy | 5 | 9 | 14 |
| Time interval between last CCRT/radiation and PET/MRI in months, median (range) | 5 (1/22) | 7 (2/55) | 6 (1/55) |
|  |  |  |  |

_____________________________

^1^TRC = treatment-related changes

^2^TPR = tumour progression or recurrence

^3^CCRT = concurrent chemoradiation therapy

^4^TMZ = temozolomide

^5^BEV =bevacizumab
