# Supplementary material for: Combined 18F-FET PET and diffusion kurtosis MRI in posttreatment glioblastoma: differentiation of true progression from treatment-related changes
Source: Neurooncol Adv. 2021 Mar 10;3(1):vdab044. doi: 10.1093/noajnl/vdab044 (PMC8117449; doi:10.1093/noajnl/vdab044)
Supplement: vdab044_suppl_Supplementary_Table_S2 [file vdab044_suppl_supplementary_table_s2.docx]

**Table 2S.** Median and quartile (Q1/Q3) values of the mean DT/KT parameters in normal-appearing white matter for the TRC (n=11) and TPR (n=21) groups and the total cohort (n=32); *p*-values of inter-group comparisons. Diffusivities are given in units of μm^2^/ms.

| Parameter | **TRC** |  | **TPR** |  |  | **Total** | |  |
| --- | --- | --- | --- | --- | --- | --- | --- | --- |
|  | Median | (Q1/Q3) | Median | (Q1/Q3) | *P*-value | Median | Q1/Q3 | |
| MD | 0.85 | (0.81/0.86) | 0.87 | (0.83/0.91) | 0.16 | 0.86 | 0.83/0.9 |  |
| RD | 0.62 | (0.61/0.63) | 0.67 | (0.61/0.73) | 0.13 | 0.64 | 0.61/0.72 |  |
| AD | 1.28 | (1.27/1.29) | 1.3 | (1.24/1.32) | 0.20 | 1.29 | 1.23/1.33 |  |
| MK | 1.0 | (0.81/1.05) | 1.04 | (0.97/1.05) | 0.74 | 1.0 | 1.0/1.1 |  |
| RK | 1.31 | (1.24/1.39) | 1.27 | (1.18/1.36) | 0.35 | 1.3 | 1.2/1.4 |  |
| AK | 0.83 | (0.78/0.87) | 0.82 | (0.80-0.87) | 0.77 | 0.8 | 0.8/0.9 |  |
